# Myricetin inhibits transmissible gastroenteritis virus replication by targeting papain-like protease deubiquitinating enzyme activity

**DOI:** 10.3389/fmicb.2024.1433664

**Published:** 2024-07-10

**Authors:** Jiahao Fan, Pengyuan Xi, Huimao Liu, Xu Song, Xinghong Zhao, Xun Zhou, Yuanfeng Zou, Yuping Fu, Lixia Li, Renyong Jia, Zhongqiong Yin

**Affiliations:** ^1^Natural Medicine Research Center, College of Veterinary Medicine, Sichuan Agricultural University, Chengdu, China; ^2^Key Laboratory of Animal Disease and Human Health of Sichuan Province, Sichuan Agricultural University, Chengdu, China

**Keywords:** myricetin, coronavirus, transmissible gastroenteritis virus (TGEV), papain-like protease (PLpro), deubiquitination

## Abstract

Myricetin, a natural flavonoid found in various foods, was investigated for its antiviral effect against transmissible gastroenteritis virus (TGEV). This α-coronavirus causes significant economic losses in the global swine industry. The study focused on the papain-like protease (PLpro), which plays a crucial role in coronavirus immune evasion by mediating deubiquitination. Targeting PLpro could potentially disrupt viral replication and enhance antiviral responses. The results demonstrated that myricetin effectively inhibited TGEV-induced cytopathic effects in a dose-dependent manner, with an EC_50_ value of 31.19 μM. Myricetin significantly reduced TGEV viral load within 48 h after an 8-h co-incubation period. Further investigations revealed that myricetin at a concentration of 100 μM directly inactivated TGEV and suppressed its intracellular replication stage. Moreover, pretreatment with 100 μM myricetin conferred a protective effect on PK-15 cells against TGEV infection. Myricetin competitively inhibited PLpro with an IC_50_ value of 6.563 μM. Molecular docking experiments show that myricetin binds to the Cys102 residue of PLpro through conventional hydrogen bonds, Pi-sulfur, and Pi-alkyl interactions. This binding was confirmed through site-directed mutagenesis experiments, indicating myricetin as a potential candidate for preventing and treating TGEV infection.

## 1 Introduction

Transmissible gastroenteritis virus (TGEV) is a single-stranded positive-sense RNA virus of the coronavirus family and the α-coronavirus genus (Lorusso et al., 2008). TGE is a highly contagious disease that affects pigs of all ages and causes transmissible gastroenteritis (TGE). TGEV infection can be associated with porcine rotavirus and porcine epidemic diarrhea virus, and is often fatal in piglets under 2 weeks of age, but rarely in adult pigs (Wang et al., 2019). The impact of TGEV infection on the global pig industry is significant, with the potential for severe economic losses (Garwes et al., 1988; Chen et al., 2010). Despite the widespread vaccination against TGEV for decades since its outbreak, highly virulent recombinant strains of TGEV continue to emerge due to frequent evolutionary and recombination events (Pensaert et al., 1993; Pritchard et al., 1999; Hu et al., 2015; Zhang et al., 2017; Guo et al., 2020; Yuan et al., 2021). The TGEV vaccine carries potential risks, including the possibility of recombination with other viruses and poor immune response. This is due to the evolutionary and recombination nature of TGEV. The natural recombinant strains JS2012 and AHHF from China are highly virulent, and viral evolution and mutation may affect vaccines and other control methods. Furthermore, the sequencing results of porcine enteric coronavirus swine enteric coronavirus (SeCoV) from European countries and canine coronavirus–human pneumonia–2018 (CCoV-HuPn-2018) virus from Malaysia indicate that these newly discovered coronaviruses are closely related to TGEV (Theze et al., 2015; Belsham et al., 2016; Boniotti et al., 2016; de Nova et al., 2020; Tortorici et al., 2022; Vlasova et al., 2022). Consequently, failure to conduct further research on TGEV could impede the public health response to transboundary outbreaks.

Porcine aminopeptidase N (pAPN) acts as the receptor for TGEV (Delmas et al., 1992). The spike protein present on the viral surface binds to PAPN, which then triggers endocytosis and internalization of the virus particles into the host cell cytoplasm (Delmas et al., 1992; Hansen et al., 1998; Tusell et al., 2007). Following viral entry, RNA is released and serves as a template for the synthesis of two replicase polyproteins (pp1a and pp1ab) from the host cell ribosome and amino acids. These polyproteins are cleaved by Nsp3 (PLP1 and PLP2) and Nsp5 (3CLpro) to generate intermediate precursors and 16 non-structural proteins (Hu et al., 2017). PLpro mediates the maturation of Nsp2| 3 and may also process the Nsp1| 2 and Nsp3| 4 cleavage sites, whose exact positions remain to be determined (Wojdyla et al., 2010).

TGEV PLpro which is comprised of PL1pro and PL2pro, is located in Nsp3 (Ziebuhr et al., 2000). So far, only the structure of TGEV PL1P has been resolved by crystallization, which is conserved among human coronavirus NL63 (HCoV-NL63) (Chen et al., 2007), mouse hepatitis virus (MHV) (Zheng et al., 2008), Middle East respiratory syndrome coronavirus (MERS-CoV) (Mielech et al., 2014), severe acute respiratory syndrome coronavirus (SARS-CoV) (Barretto et al., 2005; Lindner et al., 2005; Clementz et al., 2010), and severe acute respiratory syndrome coronavirus 2 (SARS-CoV-2) (Klemm et al., 2020). In addition to processing the replicase polyproteins, TGEV PLP1 also exhibits deubiquitinase (DUB) activity (Wojdyla et al., 2010). The innate immune response culminates in the secretion of interferon (IFN) and the upregulation of interferon-stimulated genes (ISGs), which confer an antiviral state to host cells. Ubiquitin mediates the various signaling events in this cascade and can be antagonized by the expression of viral DUB. DUB has been widely reported as a promising drug target for TGEV, coronaviruses, and other viral infections (Baez-Santos et al., 2015; Setz et al., 2017; Atkins et al., 2020).

Myricetin is a flavonol that is primarily found in the families Myricaceae, Casheaceae, Polygonaceae, Pinaceae and Primulaceae, as well as in vegetables, fruits and tea (Semwal et al., 2016). Myricetin has been shown to inhibit human immunodeficiency virus (HIV), retrovirus-like particle virus (RLV) and Moloney murine leukemia virus (MMLV) reverse transcriptases, with low cytotoxicity to host cells (Ono et al., 1990; Ono and Nakane, 1990; Chu et al., 1992). Myricetin has also been demonstrated to inhibit coronavirus replication (Xiao et al., 2021). It reduces the ATPase activity of SARS-CoV NSP13 helicase by more than 90% at a low concentration (10 μM) (Yu et al., 2012). Virtual analysis has revealed that myricetin has a high binding affinity to SARS-CoV-2 Mpro and a significant inhibitory effect on FRET. However, further investigation is required to ascertain the effect of myricetin on SARS-CoV-2 (Li et al., 2021; Xiao et al., 2021). The inhibitory effect of myricetin on coronavirus PLpro also requires improvement. Previous studies have indicated that myricetin can inhibit the deubiquitination activity of infectious bronchitis virus (IBV) PLpro and exert an antiviral effect (Peng et al., 2022). This study has confirmed the inhibitory activity of myricetin against TGEV *in vitro*. The study also evaluated the ability of myricetin to inhibit TGEV PLpro deubiquitinating enzyme activity. Furthermore, the study investigated the binding sites of myricetin with TGEV PLpro utilizing molecular docking and site-directed mutagenesis techniques. This indicated that myricetin may be a promising candidate compound for the treatment of TGEV infection.

## 2 Materials and methods

### 2.1 Cells, virus, and reagents

Swine kidney cell line (PK-15) was purchased from China Center for Type Culture Collection (Wuhan, China). PK-15 cells were propagated in Dulbecco’s modified Eagle’s medium (DMEM) supplemented with 10% fetal bovine serum (FBS), 100 U/mL penicillin, and 100 U/mL streptomycin. The cells were cultured in 5 % CO_2_ at 37°C. The Isolation methods of the TGEV strain SC2021 refer to the previously published article (Guo et al., 2020). TGEV was inoculated into PK-15 cells for proliferation and culture. The tissue culture infectious dose of 50% (TCID_50_) was 10^–6.1007^/100 μL. The plasmids pET-32a (+), and pRSFDuet-1 were purchased from Sangon Biotech Co., Ltd (Shanghai, China). The anti-hemagglutinin McAb was purchased from Proteintech Co., Ltd (Wuhan, China). Myricetin (purity > 98 %) was purchased from Chengdu Herb purify Co., Ltd (Dalian, China). The Deubiquitinase Assay Kit was purchased from Abcam (Cambridge, UK).

### 2.2 Cytotoxicity of myricetin on PK-15 cells

To determine the safe concentration ranges of myricetin for PK-15 cells, we conducted the cell counting kit-8 (CCK-8) assay. PK-15 cells were added to 96-well plates and cultured at 37°C. The medium was then replaced with DMEM containing varying concentrations of myricetin (1–1000 μM). After incubation in 5 % CO_2_ at 37°C for 48 h, 100 μL solution containing 10% CCK-8 was added to each well and incubated at 37°C for 30 min. The absorbance at 450 nm was measured with a microplate reader.

### 2.3 The antiviral effect assay

PK-15 cells were inoculated into 96-well plates and cultured for 24 h. Then wash the cells twice with serum-free DMEM and infect with 100 TCID_50_ of TGEV in each well. One hours after infection, the medium was replaced with DMEM containing various concentrations of myricetin (3.13–200 μM). After 48 h of culture, 100 μL solution containing 10% CCK-8 was added to each well and incubated at 37°C for 30 min. Absorbance at 450 nm was measured using a microplate reader. The IC_50_ value was calculated using the Reed-Muench formula (Reed and Muench, 1938): IC_50_ = C × 2^−s^ (S = N−1+(H-R)/(H-L)).

The transcription levels of the TGEV N gene were used to evaluate the antiviral effect of myricetin. The cell treatment methods used were the same as those described above. Total cellular RNA was collected at 4, 8, 12, 24, 36, and 48 h post-infection. The transcript level of the TGEV N gene was quantified using reverse transcription-polymerase chain reaction (qRT-PCR). The total RNA of the samples was prepared with the Virus Rapid DNA or RNA Kit (Biomed, Beijing, China) following the manufacturer’s instructions. The cDNA was reverse transcribed using the reverse transcription kits (Exongen, Chengdu, China). The transcription level of the Nucleocapsid (N) gene was evaluated using qRT-PCR. The qRT-PCR reaction system comprised 1 μL of cDNA template, 1 μL of a pair of specific primers (0.5 μL of the forward primer and 0.5 μL of the reverse primer), 5 μL of SYBR Green mix and 3 μL of RNA-free water. Table 1 lists the sequences of the forward and reverse primers. The qRT-PCR procedure was carried out as follows: 95°C for 5 min, followed by 40 cycles of 95°C for 10 s and 60°C for 30 s. The final extension was performed at 72°C for 60 s. The Bio-Rad CFX96*™* Manager software was used to analyze the data. The threshold cycle (CT) value was used to determine the target copy number in the reaction. The CT value corresponds to the CT value of consecutive 10-fold dilutions of a standard DNA sample.

**TABLE 1:** Primers for plasmid construction.

Specificity	Sequence (5′-3′)
Nucleocapsid	F: ATGGAGTTGTCTGGGTTGC
	R: AGAGCGTTGCTGTTGTT
Nucleocapsid for qRT-PCR	F: GGAGTTGTCTGGGTTGC
	R: TTCGCCTGGCACTTTAC
TGEV PLpro	F: GCGAGCTCTGTTCTATCCCTGTCAATGTTAC
	R: CCGCTCGAGTGCAATAGCTGTTGTGACC

### 2.4 The mode of action assay

#### 2.4.1 Pre-treatment assay

Myricetin (100 μM) was added to 6-well plates with monolayer PK-15 cells, and a mock group without myricetin was set up. Then the plates were incubated at 5% CO_2_ for 1 h at 37°C, and discard the supernatant and wash the plates three times with phosphate buffer saline (PBS). TGEV virus solution (100 μL, 10^2^ TCID_50_) was added to each well; the plates were incubated for another 1 h in the same conditions, and discard the supernatant and wash the plates three times with PBS. DMEM containing 2 % FBS was added to continue the culture for 48 h in the same conditions. The copies of the TGEV N gene in each sample were measured by absolute fluorescence quantification.

#### 2.4.2 Virus inactivation assay

Myricetin (100 μM) was mixed with TGEV virus solution (100 μL, 10^5^ TCID_50_), and a virus mock group without myricetin was set up. The mixtures were incubated for 1 h at 37°C. The mixtures were diluted 10^3^-fold and inoculated onto a 6-well plate with monolayer PK-15 cells. After that the plate was cultured for 1 h at 37°C and 5% CO_2_, and discard the supernatant and wash the plates three times with PBS. DMEM containing 2% FBS was added to continue the culture for 48 h in the same conditions. The copies of the TGEV N gene in each sample were measured by absolute fluorescence quantification.

#### 2.4.3 Inhibition of virus entry

The TGEV virus solution (100 μL, 10^2^ TCID_50_) was inoculated into a 6-well plate with monolayer PK-15 cells. Then the plate was incubated at 4°C for 1 h and discard the supernatant and wash the plates three times with PBS. Myricetin (100 μM) and a drug-free media were used as mock, and the plate was incubated at 37°C and 5% CO_2_ for 1 h before removing the supernatant. The cells were washed with citric buffer (pH 3.0) to remove the uninternalized virus. The ability of citrate buffer to affect surface charge can inhibit virus adsorption and elute uninternalized viruses (Peterson et al., 2005; Hristova and Zhivkov, 2024). Then, the cells were cultured in a maintenance medium for 48 h. The copies of the TGEV N gene in each sample were determined by absolute fluorescence quantification.

#### 2.4.4 Intracellular inhibition assay

A 6-well plate with monolayer PK-15 cells was inoculated with TGEV virus solution (100 μL, 10^2^ TCID_50_). Then the plate was incubated at 37°C and 5% CO_2_ for 1 h, and discard the supernatant and wash the plates three times with PBS. DMEM containing 2% FBS and 100 μM myricetin was added to one group of wells while DMEM containing 2% FBS without myricetin was added to another group of wells as mock, and the plate was cultured for 48 h in the same conditions. The copies of the TGEV N gene in each sample were measured by absolute fluorescence quantification.

### 2.5 Construction of recombinant plasmids

The PLpro fragment in the TGEV SC2021 genome sequence (GeneBank: ON858825.1) was used as a reference to design the specific primers of papain by using Primer 5 software. The *Sac*I restriction site was added upstream and the *Xho*I site was added downstream (marked by underlining). The reverse transcribed cDNA was used as a template to amplify the TGEV papain-like protease gene fragment by TransFast^®^ Taq DNA Polymerase (TransGen Biotech, China). The PCR reaction program was as pre-denaturation at 95°C for 30 min, denaturation at 95°C, annealing at 54°C, extension of 1 min at 72°C for 35 cycles, and extension of 10 min at 72°C. The PCR product DNA and the pET-32a (+) plasmid were digested by *Sac*I and *Xho*I enzymes, respectively. The papain gene fragment and the plasmid double enzyme fragment were recovered by a DNA purification recovery kit and ligated by T4 DNA ligase (Thermo Fisher Scientific, MA).

### 2.6 Expression of TGEV PLpro

The recombinant plasmid was transformed into the *Escherichia coli* BL21 (DE3) strain by heat shock and cultured in 2 L of fresh Luria-Bertani medium containing 50 mg/mL of ampicillin at 37°C. Once the OD_600_ reached 0.5, 0.2 mM of isopropyl β-D-1-thio-galactopyranoside was added to induce expression at 18°C for 24 h. The cells were harvested by centrifugation at 8,000 g for 3 min at 4°C. They were then suspended in lysis buffer containing 100 mM NaCl, 50 mM Tris-HCl (pH 8.0), 2 mM EDTA, and 0.5% Triton-X, and disrupted using ultrasound. After centrifugation at 12,000 g and 4°C for 20 min, the cell debris was removed. Proteins were purified using Ni-NTA agarose purification resin (Solarbio, Shanghai, China).

### 2.7 TGEV PLpro inhibition assay

The substrate Ub-AMC was selected to detect deubiquitination activity using the method (Freitas et al., 2020). The reaction was carried out in a total volume of 50 μL, consisting of 40 μL of purified TGEV PLpro, 10 μL of Ub-AMC substrate, 1 mM DTT, and various concentrations of myricetin (100, 50, 25, 12.5, 6.25, and 3.125 μM). Fluorescence intensity was continuously monitored for 30 min using a multifunctional enzyme marker with a full wavelength of Ex/Em = 350/440. The KM and Vmax values were determined by fitting the initial rate to the Michalis-Menten equation (υ = Vmax/ (1 + (Km/[S]). The fluorescence intensity curve in kinetic mode was used to fit the regression equation, and the slope of the regression equation was used to determine the enzymatic reaction rate. The change in enzymatic reaction velocity reflects the effect of deubiquitinate activity after incubation of PLpro with myricetin.

### 2.8 Western blot

Protein samples were prepared using the method in 2.6 and separated by SDS-PAGE. They were then transferred to a PVDF membrane (Milipole, USA). The membrane was sealed with 5% milk in Tris buffer solution (TBS) buffer containing 0.1% tween and incubated with the corresponding primary antibody at 4°C for 12 h. Subsequently, it was incubated with horseradish peroxidase labeled goat anti-mouse antibody or goat anti-rabbit antibody at room temperature. Electrochemiluminescence (ECL) was used to detect the reagent for developing imprinting.

### 2.9 Molecular docking

The structures of myricetin in *dsv format were downloaded from the PubChem Compound database.^1^ The ligands were hydrogenated, energy-minimized, and structure-optimized in Discovery Studio 2019 Client software. The crystal structure of TGEV SC2021 PLpro was not available in the protein database,^2^ and the homology modeling and template similarity were less than 40%. Therefore, the 3D structure of the TGEV PLpro protein was obtained from the protein database.^3^ The PLpro model structure was evaluated using the Ramachandran diagram and the Profile-3D model. To prepare the protein file in Discovery Studio 2019, water molecules were removed and hydrogen atoms were added. Molecular docking was performed using CDOCKER mode. Lastly, the protein-ligand complex results were analyzed.

### 2.10 Mutant generation

TGEV PLpro gene fragments were cloned by PCR. PLpro was connected to the pRSFDuet-1 expression vectors using the Homologous Recombination Cloning and Assembly Kit (TransGen Biotech, China). The binding sites of Myricetin with PLpro were confirmed by mutating them based on the molecular docking results. The mutant plasmids (pPLpro*^Cys102Ala^*/ pPLpro*^Arg252Ala^*/pPLpro*^His255Ala^*) were amplified using the primers listed in Table 2. The Transfast^®^ Taq DNA polymerase was used to carry out the PCR reaction according to the manufacturer’s instructions (Transgen Biotech, China). The thermal cycler program consisted of an initial denaturation at 95°C for 1 min, followed by 30 cycles at 95°C, 54°C for 1 min, and 72°C for 5 min, and finally, an extension at 72°C for 10 min. The amplified products underwent analysis through DNA agarose gel electrophoresis and were purified using the Biosharp Universal DNA purification Kit (Biosharp, China).

**TABLE 2:** Primer sequence for the mutants.

Specificity	Sequence (5′-3′)
pPLpro^Cys102Ala^	F: CATTAATCCAAGCGTTGTTGTCAGTC
	R: CAACGCTTGGATTAATGCAATTTGTCTTG
pPLpro^Arg252Ala^	F: GACCATTAGCATTAGAACCGCTATAAC
	R: CTAATGCTAATGGTCATTACACCTATTACG
pPLpro^His255Ala^	F: GTGTAAGCACCATTCCTATTAGAACCG
	R: GAATGGTGCTTACACCTATTACGATAACC

### 2.11 Statistical analysis

Statistical analysis was conducted using IBM SPSS 21 software. The significance of the data was established using the student’s *one-way ANOVA*. GraphPad Prism 9 software was used to visualize the data, and statistically significant differences were set at *P* < 0.05 (*), *P* < 0.01 (**), and *P* < 0.001 (***).

## 3 Results

### 3.1 Cytotoxicity of myricetin on PK-15 cells

**FIGURE 1:**
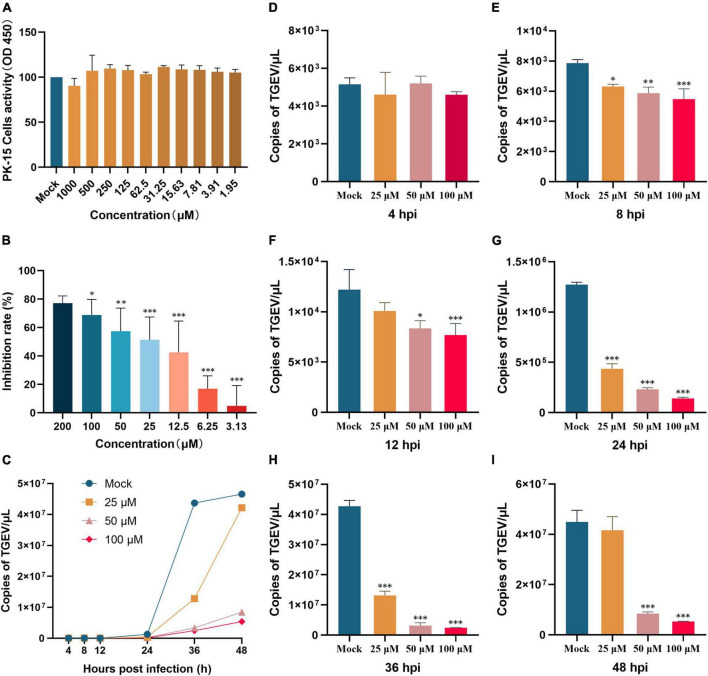
Myricetin inhibits TGEV infection and affects viral growth in PK-15 cells. **(A)** Cell viability of PK-15 cells treated with various concentrations of myricetin (0–1000 μM) for 48 h. **(B)** PK-15 cells were infected with TGEV and treated with different concentrations of myricetin for 24 h. The inhibition rate of TGEV infection was calculated by comparing the percentage of cell viability in treated cells to that of untreated cells. Results showed that myricetin inhibited TGEV infection in a dose-dependent manner. **(C)** Effects of different concentrations of myricetin on TGEV growth curve. Real-time fluorescence quantification assays were performed to observe the TGEV N gene copies. The y-axis represents the copies, and the x-axis indicates myricetin treatment time. **(D–I)** Copies of the TGEV N gene were measured at 4, 8, 12, 24, 36, and 48 h after infection. The terms “Mock”, “25 μM”, “50 μM”, and “100 μM” refer to different treatments with myricetin at different concentrations, including no treatment (0 μM), 25, 50, and 100 μM, respectively. Data are presented as mean ± SD of three independent experiments. The different symbols on the columns represent the significant differences compared with mock group (*P < 0.05; **P < 0.01; ***P < 0.001).

The CCK-8 assay was used to assess the cytotoxicity of myricetin in PK-15 cells. Myricetin treatment at various concentrations (0–1000 μM) did not significantly (*P* > 0.05) affect the viability of PK-15 cells, indicating that PK-15 cells are safe when exposed to myricetin concentrations below 1000 μM (Figure 1A).

### 3.2 Myricetin attenuates TGEV-induced cytopathic effects in PK-15 cells

Cytopathic effects of TGEV on PK-15 cells were dose-dependently attenuated by myricetin PK-15 cells were infected with 100 TCID_50_ TGEV and treated with 0–200 μM myricetin for 48 h. Cell viability was measured by CCK-8 assay. As shown in Figure 1B, myricetin at concentrations lower than 12.5 μM did not significantly inhibit the TGEV, whereas myricetin at 200 μM inhibited it by 78%. The EC_50_ value of myricetin against TGEV-infected PK-15 cells was 31.19 μM, as calculated by the Reed-Muench method.

### 3.3 The antiviral activity for myricetin against TGEV

To investigate the inhibitory effect of myricetin on TGEV activity, an absolute fluorescence quantitative detection method was constructed using the TGEV N gene as a template. We constructed pcDNA3.1-N recombinant plasmid and used the 10-fold gradient diluted recombinant plasmid as a template for the qRT-PCR reaction. After amplification, Cq values were determined for each sample. The standard curve was plotted with the logarithm of the plasmid concentration as the horizontal coordinate and the Cq value as the vertical coordinate: *Y* = −3.594X+42.439. The TGEV copies (TGEV N gene transcription level) were then assessed at 4, 8, 12, 24, 36, and 48 h post-infection. As shown in Figures 1C–I, there was no significant difference in TGEV copies between the mock group and the groups treated with 100, 50, or 25 μM myricetin at 4 h, which might reflect the latency period of the virus and the lack of substantial increase in virus copies. By contrast, at 8, 12, 24, and 36 h after infection, TGEV copies were significantly lower in the myricetin-treated groups than in the mock group (*P* < 0.01). Interestingly, at 48 h after infection, a rebound in the number of TGEV copies was observed in the group treated with 25 μM myricetin, which was not significantly different from the mock group. We hypothesize that this is due to the low concentration of myricetin and its weak inhibitory effect at longer virus growth times.

**FIGURE 2:**
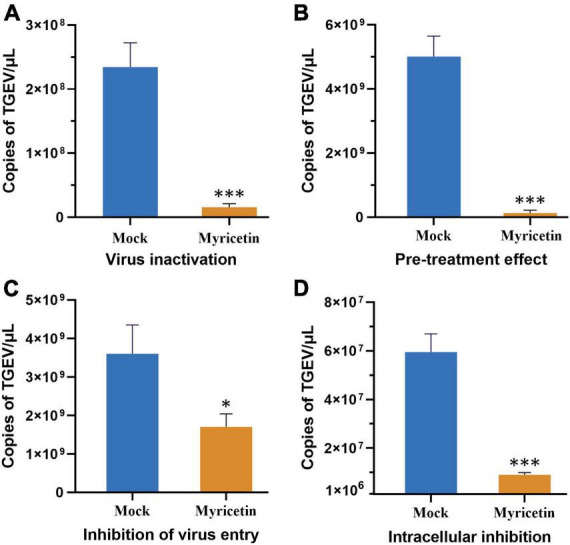
Effects of different modes of action of myricetin on TGEV growth. **(A)** The direct killing effect of myricetin on TGEV. **(B)** The preventive effect of myricetin on TGEV. **(C)** The inhibitory effect of myricetin on TGEV penetration. **(D)** Inhibitory effect of myricetin on intracellular replication of TGEV. Mock and myricetin refer to treatments with 0 and 100 μM myricetin, respectively. Data are presented as mean ± SD of three independent experiments. Significant difference analysis: No significant difference: *P* > 0.05, *P < 0.05, ***P < 0.0001.

Changes in TGEV copies were also measured by administering myricetin in different ways. A direct killing effect on TGEV by 100 μM myricetin was found (Figure 2A), and virus copies after myricetin treatment were significantly lower than those in the mock group (*P* < 0.001). As shown in Figure 2B, the preventive treatment group also showed a significant difference in virus copies compared with the mock group (*P* < 0.001), suggesting that myricetin could protect the cells. Moreover, myricetin could significantly inhibit the process of TGEV entry and intracellular replication (Figures 2C, D). Therefore, myricetin had antiviral activity in the direct killing effect, preventive effect, and the process of virus entry and intracellular replication.

### 3.4 Inhibition of TGEV PLpro deubiquitinating activity by myricetin

**FIGURE 3:**
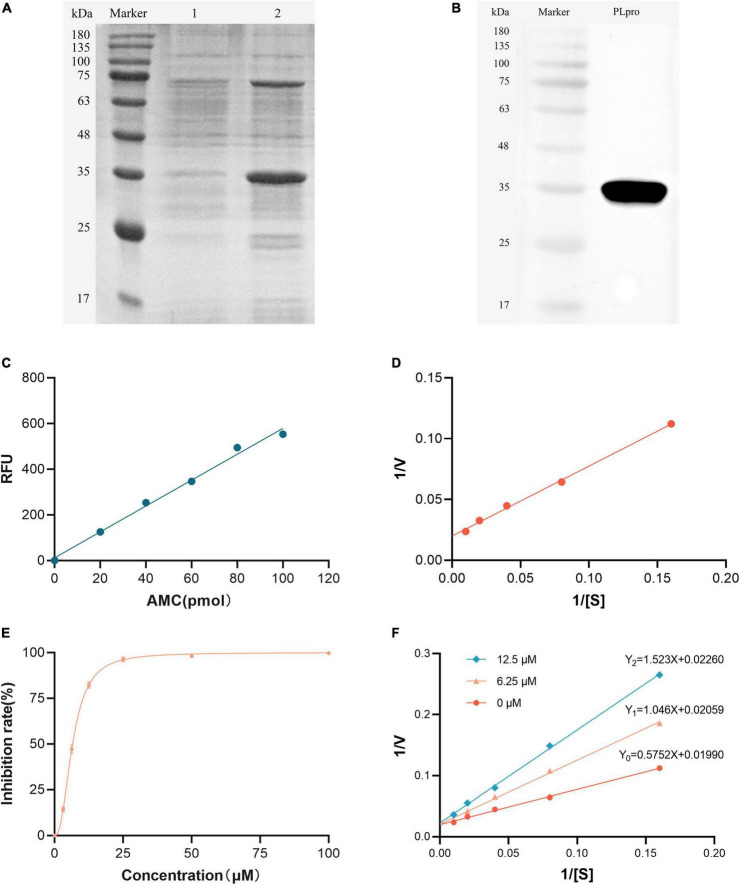
Inhibition of TGEV PLpro deubiquitinating activity by myricetin. **(A)** SDS-PAGE analysis of *E. coli* BL21 cells transformed with pET-32a-PLpro. Lane 1 represents uninduced cells while lane 2 represents induced cells. **(B)** Western blot of the purified TGEV PLspro protein using anti-His tag antibody. **(C)** AMC standard curve. The fluorescence intensity (Y) had a linear relationship with the AMC content (X) (*Y* = 5.8328X). **(D)** Double reciprocal plot for the detection of TGEV PLpro activity. The initial enzyme velocity (V) was inversely proportional to the substrate concentration ([S]) according to the Michaelis-Menten equation. The slope and intercept of the plot were utilized to compute the enzyme-substrate affinity (Km) and the maximum enzyme reaction velocity (Vmax). **(E)** Inhibition rate of myricetin at different concentrations on PLpro deubiquitinating enzyme activity. Myricetin inhibited TGEV PLpro activity in a dose-dependent manner, with an IC_50_ of 6.563 μM. **(F)** Double reciprocal plot for the inhibition of TGEV PLpro by different concentrations of myricetin. Myricetin increased the Km of TGEV PLpro without affecting the Vmax, indicating a competitive inhibition mode.

A recombinant expression vector for the PLpro of TGEV was constructed. The TGEV PLpro gene was cloned into the pET-32a (+) vector and the recombinant plasmid was verified by double enzyme digestion and sequencing. The recombinant PLpro protein was expressed in *E. coli* BL21 and purified by affinity chromatography. The purified PLpro protein was confirmed by SDS-PAGE and Western blotting (Figures 3A, B).

The activity of TGEV PLpro was measured using the Abcam Deubiquitinase Assay Kit. 7-Amino-4-methylcoumarin (AMC), a fluorophore modified at the C-terminus of the peptide, was used as a substrate for PLpro. The AMC standard curve was plotted by measuring the fluorescence values of different concentrations of AMC, and a good linear relationship was found between the AMC content and the fluorescence intensity value (*Y* = 5.8328X, R^2^ = 0.9971, Figure 3C). Ubiquitin-AMC (Ub-AMC) was used as the enzyme substrate and fluorescence accumulation was monitored over time as PLpro cleaved Ub-AMC and released AMC. The initial enzyme velocity of TGEV PLpro at different substrate concentrations was obtained by continuous fluorescence detection, and the enzyme-substrate affinity (Km = 28.9045) and the maximum enzyme reaction velocity (Vmax = 50.2513 pmol/min/mL) were calculated according to the Michaelis-Menten equation (Figure 3D). The inhibitory effect of myricetin on TGEV PLpro activity was next tested. TGEV PLpro was co-incubated with myricetin solutions at different concentrations (100, 50, 25, 12.5, 6.25, and 3.125 μM) in the dark and the initial enzyme reaction rate was measured for each concentration (Figure 3E). It was found that myricetin had no apparent inhibitory effect on TGEV PLpro at 3.125 μM, but the enzyme activity was reduced by almost 50 % at 6.25 μM and by 100% at 25 μM. The half-maximal inhibitory concentration (IC_50_) of myricetin against TGEV PLpro was calculated to be 6.563 μM. PLpro was co-incubated with myricetin solutions at 12.5, 6.25, and 0 μM and different concentrations of substrate mix were added to start the enzyme reaction. The double reciprocal curve equation was plotted with 1/[S] as the horizontal axis and 1/V as the vertical axis for each myricetin concentration group. The regression equations were Y_0_ = 0.5752X + 0.01990 (R^2^ = 0.9976), Y_1_ = 1.046X + 0.02059 (R^2^ = 0.9975), and Y_2_ = 1.523X + 0.02260 (R^2^ = 0.9989), respectively. It was observed that myricetin did not alter the Vmax of papain but increased the Km, indicating that myricetin exerts a competitive inhibition on papain (Figure 3F).

### 3.5 Molecular docking analysis of the binding site of myricetin and TGEV PLpro protein

**FIGURE 4:**
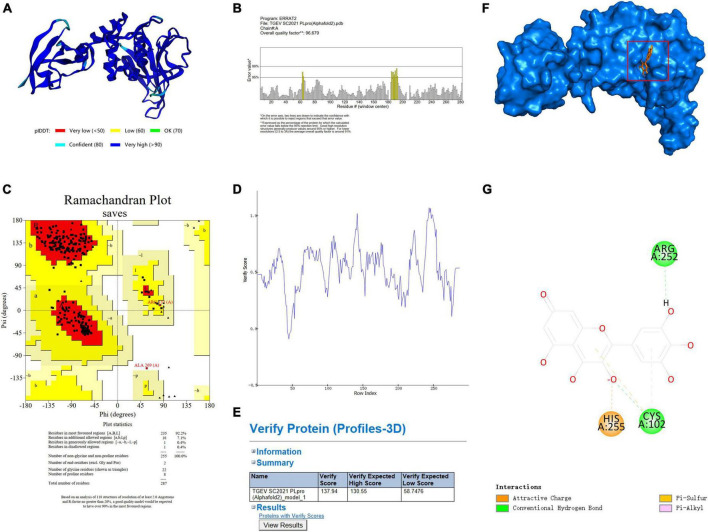
TGEV PLpro three-dimensional model establishment and analysis. **(A)** The 3D structure of PLpro has been determined by using Alphafold2. **(B)** The Errat2 model is used to calculate scores for amino acid residues. **(C)** Ramachandran plots show regions permitted for the homologous mode of PLpro. The regions are color-coded; Red (best), Yellow (appropriate), Light yellow (barely permitted), and White (disallowed). **(D,E)** 3D plot profile for the PLpro protein structure. **(F)** Ligand docking model of PLpro from TGEV SC2021 and myricetin. The blue surface represents the crystal structure of PLpro from TGEV while the orange stick model represents myricetin. **(G)** The 2D interactions between myricetin and PLpro involve orange dotted lines (attractive charge), light orange dotted lines (Pi-sulfur), green dotted lines (conventional hydrogen bond), and pink dotted lines (Pi-alkyl).

Molecular docking was used to predict and visualize the interaction between TGEV PLpro and myricetin. However, the crystal structure of TGEV SC2021 PLpro remains unsolved, and the homology model constructed has less than 40% sequence identity with the template. Therefore, Alphafold2, an online protein structure prediction server, was employed to model TGEV SC2021 PLpro (Figure 4A). Most amino acid residues have very high confidence (>90 %, dark blue), a few have high confidence (80 %, blue), and the overall model quality score is 96.679 (Figure 4B). The X-ray structure was evaluated using the Errat evaluation programme, the Ramachandran Plot in the Procheck evaluation programme, and the Profile-3D model module of the Discovery Studio software.

Figure 4C shows the TGEV PLpro residues in the Ramachandran Plot. According to the analysis of phi and psi angles of PLpro residues, it was found that 92.2 % (235 residues) of them were in the most favored region, 7.1 % (18 residues) were in the additional allowed region, 0.4 % (1 residue) were in the generously allowed region, and 0.4 % (1 residue) were in the disallowed region. The majority of PLpro residues exhibit energetically favorable torsion angles, as shown in Figure 4C. The optimal region is represented by the red area in the Ramachandran plot. This suggests that the modeled structure conforms to the crystal structure of TGEV PLpro, implying that the protein structure is plausible. We used the model evaluation program, Profiles-3D, to assess the quality of packing for each residue. The reliability of the model increases with the verification score of the amino acid residues (Denison et al., 1992). As shown in Figures 4D, E, the verification scores of all amino acid residues were positive and the comprehensive score was 134.97. Therefore, the model structure is reasonable and suitable for molecular docking.

The lowest energy score (CDOCKER energy −44.0536 kcal/mol; CDOCKER interaction energy −40.7904 kcal/mol) was chosen to demonstrate the binding of myricetin and PLpro. The docking pattern in three dimensions, along with a schematic representation of planar amino acid residues, indicates that myricetin interacts with three amino acids (Cys102, Arg252, and His255) in the active site of PLpro. This suggests that these three amino acids are potential sites for the interaction of myricetin with PLpro. Myricetin binds to PLpro Cys102 by conventional hydrogen bond, Pi-sulfur and Pi-alkyl interactions, to Arg 252 by conventional hydrogen bond, and to His255 by charge attraction (Table 3). These results indicate that the potential binding sites of myricetin to PLpro are Cys102, Arg 252, and His 255.

**TABLE 3:** Parameters of interaction bond formation between Myricetin and TGEV PLpro.

Donors atom	Receptor atom	Distances(Å)	Interactions
Cys102	Myricetin O	2.41	Conventional hydrogen bond
Cys102		4.88	Pi-sulfur
Cys102		4.50	Pi-alkyl
Arg252	Myricetin O	1.98	Conventional hydrogen bond
His255		4.71	Attractive charge

### 3.6 Myricetin was insensitive to Cys102 mutants

**FIGURE 5:**
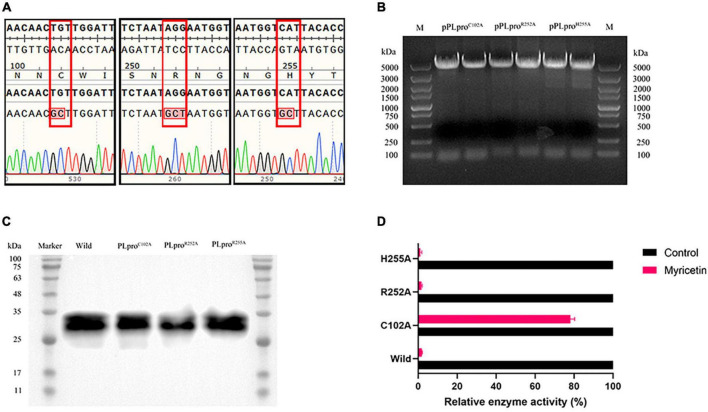
Inhibitory activity of myricetin on mutant TGEV PLpro. **(A)** The mutant plasmid DNA sequencing results are arranged from left to right as follows; C102A, R252A, and H255A respectively. The results showed that all three mutation sites were alanine. **(B)** Identification of pRSFDuet-1-PLpro recombinant plasmid for TGEV PLpro. M: 5 kb DNA Marker. **(C)** Recombinant PLpro western blot results. **(D)** Inhibition rate of myricetin on TGEV PLpro mutant protein. C102A mutation can increase the relative enzyme activity of PLpro.

Three recombinant plasmids were synthesized by site-directed mutagenesis (pPLpro^C102A^, pPLpro^R252A^ and pPLpro^H255A^) and verified by DNA sequencing (Figures 5A, B). The original plasmid and the mutant plasmids of PLpro were transferred into *E. coli* BL21 and the wild type PLpro and the mutants PLpro^C102A^, PLpro^R252A^ and PLpro^H255A^ were induced and expressed (Figure 5C). The inhibitory activity of myricetin on the wild-type and the mutant PLpros was determined by using a Deubiquitinase Assay Kit. The inhibition rates of myricetin on PLpro^R252A^, PLpro^H255A^ and wild-type PLpro activity were 98.48 %, 98.39 % and 99.65 %, respectively, whereas the inhibition rate of myricetin on PLpro^C102A^ was only 19.41 %, implying that the binding site of myricetin to TGEV PLpro is Cys 102 (Figure 5D).

## 4 Discussion

Herbs extracted from Chinese plants have been traditionally used to treat various infections and diseases. A class of flavonoids derived from Chinese herbal medicine (CHM) is being considered as potential protective agent for the novel coronavirus (nCoV) (Khazdair et al., 2021). Myricetin has been demonstrated to exhibit inhibitory activity on SARS-CoV-2 Mpro, preventing the virus from entering the host cell by blocking the binding of the spike protein receptor-binding domain (RBD) to the angiotensin-converting enzyme 2 (ACE2) receptor (Xiao et al., 2021; Pan et al., 2023). Previous studies have demonstrated that PLpro is a conserved cysteine protease in coronaviruses, which is capable of cleaving virus replicase polymers and inhibiting the host innate immune response through deubiquitination and deISGylation activities (Mielech et al., 2015; Freitas et al., 2020). Consequently, the inhibition of PLpro may not only impede virus replication but also enhance the host’s antiviral response. Previous studies conducted in our laboratory have demonstrated that myricetin can suppress IBV PLpro deubiquitination activity, thereby inhibiting IBV replication (Peng et al., 2022). Nevertheless, the impact of myricetin on transmissible gastroenteritis virus (TGEV) and PLpro remained uncertain. In this study, we demonstrated that myricetin can impede the growth and replication of TGEV by targeting its PLpro. Our findings indicate that myricetin may be a prospective anti-TGEV compound and offer novel insights into the development of antiviral drugs against coronaviruses.

The cytotoxicity and therapeutic effect of myricetin on TGEV-infected cells were evaluated. Many antiviral drugs have been eliminated from further consideration due to high cytotoxicity in the screening process (De Clercq, 2004). A previous study indicated that the concentration of myricetin required to achieve a 50 % reduction in cell viability in A549, THP1, and HEK293 cells was greater than 400 mM (Pan et al., 2023). The results demonstrated that myricetin did not exhibit overt cytotoxicity at the concentration of 1000 μM, indicating a high safety range for myricetin, consistent with previous reports. Following the administration of myricetin to TGEV-infected PK-15 cells for 48 h, the cell viability was observed to be restored to a level comparable to that of uninfected cells. This indicated that the therapeutic dose of myricetin was 22.27 μM. In addition to the coronavirus, myricetin has been reported to inhibit the proliferation of other viruses, including the Zika virus (ZIKA), herpes simplex virus (HSV) and pseudorabies virus (PRV), *in vitro*. In a previous study, myricetin was demonstrated to inhibit ZIKA entry into Vero cells using virus plaque and RNA tests, with an EC_50_ of only 0.58 ± 0.17 μM (Zou et al., 2020). In the *in vitro* inhibition experiment of the pseudorabies virus, the IC_50_ of myricetin on PRV was 42.69 μM (Hu et al., 2022). Interestingly, in a previous study, it was found that myricetin had an EC_50_ of 5 μM against the HSV-1 virus and no significant inhibitory effect on HSV-2 (Lyu et al., 2005), while in another study it was found that the EC_50_ values of myricetin on HSV-1 and HSV-2 were 2.2 ± 0.3 μM and 1.6 ± 0.5 μM, respectively (Li et al., 2020). Flavonoids may interfere with the life cycle of coronavirus (Russo et al., 2017; Spagnuolo et al., 2018), which can be divided into adsorption, penetration, intracellular reproduction and release stages. To investigate the impact of myricetin on TGEV life cycle inhibition, we quantified the replication of TGEV N genome in PK-15 cells, reflecting the effect of myricetin treatment on different stages of TGEV infection. Our findings demonstrated that myricetin could directly inactivate TGEV and block its entry and replication in PK-15 cells, a porcine kidney cell line. Furthermore, myricetin conferred protection to PK-15 cells against TGEV infection. Furthermore, myricetin demonstrated a dose-dependent inhibition of TGEV copy numbers, with the strongest inhibition observed at 24–36 h post-infection. Notably, at 48 h post-infection (48 hpi), the viral copy number of the 25 μM myricetin treatment group returned to a level comparable to that of the mock group, in contrast to the results observed in the EC_50_ assay. The 25 μM myricetin-treated group was able to significantly suppress viral copies at the early stage, while viral copies rebounded to be no different from the mock group at 48 h. We hypothesized that myricetin may have protected the infected PK-15 cells at the early stage by inhibiting the key proteins of TGEV.

PLpro gene exists in the first ORF of coronavirus, which could be translated into two polyproteins, pp1a and pp1ab, and then co-processed by Mpro and PLpro into 16 NSPs (Cui et al., 2019; Dai et al., 2020). Virus infection activates the host’s innate immunity, which plays an antiviral role in early infection (Chhabra et al., 2016). The ubiquitination of innate immune proteins is a key factor in their activity, and the deubiquitination of PLpro by PLpro impedes this process. Myricetin was tested for its inhibition and type on TGEV PLpro activity, and it was found to inhibit PLpro by more than 75 %, with an IC_50_ of 6.563 μM. Three concentrations of 12.5, 6.25, and 0 μM were employed in the experiment, and it was found that myricetin treatment did not alter the maximum velocity of the enzyme (Vmax), but did increase the Michaelis constant (Km). According to the Michaelis equation, myricetin competitively inhibits the papain-like protease.

Molecular docking visualizes receptor-ligand interaction (Joshi et al., 2020). We predicted myricetin’s binding sites on TGEV PLpro by docking. Myricetin was not covalently bound to Cys102/Arg252/His255 but was closely bound to PLpro by higher interaction energy. However, docking accuracy and reliability may be affected by receptor and ligand conformation and flexibility. The core region of PLpro catalysis is a conservative Cys-His-Asp triad (Han et al., 2005), similar to our prediction, except for Arg252. To verify our results, we assessed the impact of myricetin on the activity of the PLpro enzyme after inducing mutations in Cys102, Arg252, and His255. Only PLpro^C102A^ was not significantly inhibited by myricetin (19.41 %), while PLpro^R252A^ and PLpro^H255A^ had similar inhibition rates as wild-type PLpro. We speculated that the static molecular docking was different from the actual dynamic binding environment.

In summary, our results show that myricetin can bind to TGEV PLpro Cys102, competitively inhibit the activity of TGEV PLpro deubiquitination, directly inhibit TGEV when co-incubated with TGEV, and protect PK-15 cells by inhibiting the entry and intracellular replication of TGEV virions of TGEV-infected cells.

## 5 Conclusion

In conclusion, targeting TGEV PLpro can be an effective strategy for designing antiviral agents and the antiviral effects of myricetin on TGEV are achieved through the inhibition of the deubiquitinating activity of PLpro Cys102.

## Data availability statement

The raw data supporting the conclusions of this article will be made available by the authors, without undue reservation.

## Ethics statement

Ethical approval was not required for the studies on animals in accordance with the local legislation and institutional requirements because only commercially available established cell lines were used.

## Author contributions

JF: Conceptualization, Formal analysis, Investigation, Methodology, Resources, Data curation, Validation, Visualization, Writing-original draft. PX: Conceptualization, Formal analysis, Investigation, Methodology, Resources, Data curation, Validation, Visualization, Writing-original draft, Writing –review and editing. HL: Visualization, Formal analysis, Investigation, Methodology, Writing – review and editing. XS: Validation, Visualization, Writing – review and editing. XHZ: Conceptualization, Formal analysis, Funding acquisition, Investigation, Methodology, Project administration, Resources, Supervision, Writing – review and editing. XZ: Validation, Visualization, Writing – review and editing. YZ: Validation, Visualization, Writing – review and editing. YF: Validation, Visualization, Writing – review and editing. LL: Validation, Visualization, Writing – review and editing. RJ: Visualization, Formal analysis, Investigation, Supervision, Writing – review and editing. ZY: Conceptualization, Formal analysis, Funding acquisition, Investigation, Methodology, Project administration, Resources, Supervision, Writing – review and editing.

## Footnotes

1
https://www.ncbi.nlm.nih.gov/pccompound

2
https://www.rcsb.org

3
https://colab.research.google.com/github/deepmind/alphafold/blob/main/notebooks/AlphaFold.ipynb
